# Pilot Trial of a Noninvasive Closed-Loop Neurotechnology for Stress-Related Symptoms in Law Enforcement: Improvements in Self-Reported Symptoms and Autonomic Function

**DOI:** 10.1177/2164956120923288

**Published:** 2020-05-07

**Authors:** Catherine L Tegeler, Hossam A Shaltout, Sung W Lee, Sean L Simpson, Lee Gerdes, Charles H Tegeler

**Affiliations:** 1 Department of Neurology, Wake Forest School of Medicine, Winston-Salem, North Carolina; 2 Hypertension and Vascular Research Center, Wake Forest School of Medicine, Winston-Salem, North Carolina; 3 University of Arizona School of Medicine, Phoenix, Arizona; 4 Department of Biostatistical Sciences, Wake Forest School of Medicine, Winston-Salem, North Carolina; 5 Brain State Technologies, Scottsdale, Arizona

**Keywords:** neurotechnology, heart rate variability, law enforcement, HIRREM, insomnia, stress

## Abstract

**Background:**

Law enforcement officers have decreased life expectancy, attributed to work-related exposure to traumatic stress and circadian disruption. Autonomic dysregulation is reported with traumatic stress and chronic insomnia.

**Objective:**

We explore potential benefits for reduced symptoms related to stress and insomnia and improved autonomic function associated with open label use of high-resolution, relational, resonance-based, electroencephalic mirroring (HIRREM®), in a cohort of sworn law enforcement personnel.

**Methods:**

Closed-loop noninvasive therapies utilizing real-time monitoring offer a patient-centric approach for brain-based intervention. HIRREM® is a noninvasive, closed-loop, allostatic, neurotechnology that echoes specific brain frequencies in real time as audible tones to support self-optimization of brain rhythms. Self-report symptom inventories done before and after HIRREM included insomnia (ISI), depression (CES-D), traumatic stress (PCL-C), anxiety (GAD-7), perceived stress (PSS), and quality of life (EQ-5D). Ten-minute recordings of heart rate and blood pressure allowed analysis of baroreflex sensitivity (BRS) and heart rate variability (HRV).

**Results:**

Fifteen participants (1 female), mean (SD) age 45.7 (5.6), received 12.2 (2.7) HIRREM sessions, over 7.9 in-office days. Data were collected at baseline, and at 22.8 (9.2), and 67.2 (14.1) days after intervention. All symptom inventories improved significantly (*P* < .01), with durability for 2 months after completion of the intervention. The use of HIRREM was also associated with significant increases (*P* < .001) in HRV measured as rMSSD and BRS measured by high-frequency alpha index. There were no serious adverse events or drop outs.

**Conclusion:**

These pilot data provide the first report of significant symptom reductions, and associated improvement in measures of autonomic cardiovascular regulation, with the use of HIRREM in a cohort of law enforcement personnel. Randomized clinical trials are warranted.

## Background

A major challenge for law enforcement officers is their near continuous exposure to stressors, putting them at significant risk for compromises in their health and performance. A report from the Buffalo Cardiometabolic Occupational Police Stress (BCOPS) study found that for white male police officers in Buffalo, New York average life expectancy was 21.9 years lower than for the white male general US population.^1^ Among potential causes for the discrepancy, the authors cited repeated stress exposures, including an estimated 7% to 19% prevalence of posttraumatic stress disorder (PTSD), and also biological circadian rhythm disruption due to shift work. Similarly, in a review of cardiovascular health risks in law enforcement, Zimmerman pointed to physical and psychological stresses including shift work, as being risk factors for hypertension, dyslipidemia, obesity, and diabetes.^2^

Importantly, sleep disturbance is highly associated with exposure to chronic stress^3^ and trauma,^4^ and evidence shows that sleep, as such, is a critical factor in the health and performance of individuals working in high stress environments. Gehrman et al. found that among military service members deployed to the Middle East after the 9/11 terror attacks, predeployment insomnia symptoms were a significant risk factor for postdeployment diagnosis of PTSD. Interestingly, the increased risk for subsequent PTSD related to predeployment symptoms of insomnia was almost as strong as their measure for combat exposure.^5^ Hartley et al. noted that the percent of police officers with depression was roughly double that for the general population.^6^ It was reported that officers in this study were almost 4 times more likely to get less than 6 hours of sleep in a given 24-hour period than the general population. Furthermore, a study of US and Canadian police officers found that 40% screened positive for at least 1 sleep disorder (sleep apnea, insomnia, or shift work disorder). Those who screened positive had higher odds to report that they had made a serious administrative error, fallen asleep while driving, made an error or safety violation related to fatigue, had uncontrolled anger toward suspects, or fallen asleep during meetings.^7^ The National Law Enforcement and Corrections Technology Center also found that over half of officers fail to maintain healthy sleep patterns and 90% report being routinely fatigued on the job.^8^

Neurobiological pathways are believed to mediate or amplify the effects of stress, circadian rhythm disruption, and sleep disturbance on health and performance. This includes both the functionality of the neuroendocrine axis^9^ and the autonomic nervous system. In particular, interactions between the sympathetic (“fight-flight”) and parasympathetic (“rest-digest-freeze”) divisions are of interest.^10^ Dysregulated activity of the sympathetic and parasympathetic divisions are increasingly a focus for studies involving stress in law enforcement officers. These may be expressed as a dampening or decrease of heart rate variability (HRV) or baroreflex sensitivity (BRS). HRV, or variability in the beat-to-beat interval between heart contractions, indicates the capacity of the cardiovascular system to make dynamic adjustments to cardiac output, in the context of instantaneous and anticipated environmental needs. In general, greater HRV and BRS indicate a higher capacity for the parasympathetic division to modulate or buffer the arousal of the sympathetic division, while lower HRV is a risk factor for adverse health and performance outcomes.^11^,^12^ In the BCOPS study, HRV was shown to vary inversely with insulin levels in a cross-sectional analysis (n = 355) of nondiabetic police officers.^13^ The same team found that HRV was inversely related to the exposure of perceived lack of support, in female officers.^14^

These data indicate that stress, sleep disturbance, and autonomic dysregulation present a major challenge to the health, well-being, and operational performance of law enforcement officers. As yet there is no evidence pointing to robust, acceptable, brief, durable, and cost-effective interventional strategies for helping law enforcement personnel to simultaneously optimize their sleep quality and attenuate adverse tendencies in autonomic nervous system activity. Given its central role with sleep, and with managing autonomic responses to traumatic stress, brain-focused approaches appear to offer an attractive target for therapeutic interventions.

High-resolution, relational, resonance-based, electroencephalic mirroring (HIRREM^®^) is a noninvasive, closed-loop, allostatic, brain echoing neurotechnology developed, registered to, and licensed by Brain State Technologies, LLC, Scottsdale, Arizona, to support autocalibration of neural oscillations. Scalp sensors observe brain frequencies and amplitudes in real time, and software-guided algorithms identify and translate selected brain frequencies into audible tones to support real-time self-optimization of brain activity.^15^ The audible tones are echoed back to the recipient via ear buds bilaterally, simultaneously, in 4 to 8 milliseconds. This provides an opportunity for the recipient to, figuratively speaking, listen to their brain. The rapid updating regarding its own pattern, and resonance between the audible tones and oscillating brain networks, provides the brain a chance to auto-calibrate, self-adjust, “relax,” and reset/get unstuck from what have been persisting stress/trauma response patterns. The brain electrical patterns are observed to shift independently, with no conscious, cognitive activity required, no operant conditioning, and no learner in the loop, toward improved balance and reduced hyperarousal. Such acoustic neuromodulation with the associated increase in flexibility and dynamic range of response might be expected to translate into health benefits such as reduced symptoms and improved downstream autonomic cardiovascular regulation.

The use of HIRREM has been associated with reduced sleep symptomatology and reduced high-frequency amplitudes in adults with insomnia,^16^ reduced menopausal symptoms in women,^17^ improved sleep in athletes with persisting postconcussion symptoms,^18^ reduced symptoms, and temporal lobe high-frequency asymmetry in self-reported posttraumatic stress,^19^ and reduced symptoms of military-related traumatic stress.^20^ Improved autonomic cardiovascular regulation has been observed in a large heterogeneous cohort of those receiving HIRREM.^21^ In addition, correlation has been reported between high-frequency electrical brain pattern asymmetry scores at baseline and measures of autonomic cardiovascular regulation.^22^ Moreover, improved network connectivity on whole-brain rest magnetic resonance imaging was observed in participants with military-related traumatic stress.^23^ Finally, in a controlled trial of HIRREM for moderate to severe insomnia (auditory tones linked to brainwaves vs random tones), there was not only added benefit with the active intervention to reduce symptoms, but significant increases were also observed in multiple objective measures of autonomic cardiovascular regulation, with durability to 4 months post-HIRREM.^24^,^25^ Based on previous experience, the hypothesis was that the use of HIRREM by law enforcement personnel would be associated with reduced symptoms and improved autonomic function. The current report explores the effects of the open label use of HIRREM on autonomic cardiovascular regulation and self-reported symptoms in a cohort of law enforcement personnel.

## Methods

### Population and Subject Recruitment

This single site, exploratory, open label, institutional review board (IRB)-approved study was conducted by the Department of Neurology at Wake Forest School of Medicine, Winston-Salem, North Carolina, USA. The parent study included individuals who had at least 1 or more diverse neurological, cardiovascular, or psychophysiological conditions. Participants for this case series were drawn from among those who enrolled between March 17, 2017 and September 29, 2017. These subjects were recruited from local law enforcement branches through in-service presentations, by self-referral, and after word of mouth from other participants. Those who expressed interest were screened for eligibility through an online questionnaire followed by e-mail or telephone communication. Written, informed consent was obtained from all participants.

Criteria for inclusion in this cohort included being a sworn law enforcement officer. These participants were recruited as a convenience cohort by posting and distributing IRB-approved flyers, and by word of mouth, and were all self-referred. Exclusion criteria included the inability to attend study visits and provide informed consent, sit in a chair comfortably, and refrain from using recreational drugs, alcohol, or energy drinks during the intervention and for a month following the intervention. Those with bilateral total hearing loss, known seizure disorders, or ongoing use of benzodiazepines, opiate, or antipsychotic medication were also excluded.

### Study Timeline

After providing informed consent, participants completed baseline (visit 1, V1) outcome measures (details later), including self-report symptom inventories, and measures, along with an assessment of brain electrical activity. Participants then received a series of HIRREM sessions, a post-HIRREM in-office data collection (visit 2, V2), and a late in-office data collection (visit 3, V3). Participants were asked to continue with their current medical and behavioral care throughout the study.

### Outcome Measures

#### Self-reported symptom inventories

Outcome measures were administered to evaluate symptoms related to stress such as sleep difficulty, depressive mood, anxiety, traumatic stress, and quality of life. Sleep was evaluated using the Insomnia Severity Index (ISI). This 7-item measure is scored on a 5-point Likert-type scale from 0 (no problem) to 4 (very severe problem).^26^ Total scores can range from 0 to 28, reflecting the degree of insomnia over the previous 2 weeks: absence (0–7), subthreshold (8–14), moderate (15–21), and severe (22–28). Score reduction of at least 6 to 7 points represents the minimum change for clinical difference with insomnia symptoms.^27–29^

Depressive mood was assessed using the Center for Epidemiologic Studies Depression scale (CES-D).^30^ This 20-item survey focuses on symptoms over the previous week, with scores ranging from 0 to 60. A score of 16 or greater is considered as a threshold to suggest clinical depression.^31^

Traumatic stress was evaluated with the Posttraumatic Stress Disorder Checklist—Civilian version (PCL-C) which includes PTSD Criteria B, C, and D from the American Psychiatric Association’s Diagnostic and Statistical Manual of Mental Disorders.^32^ This measure addresses symptoms present over the previous month that may pertain to any traumatic life experience. It includes 17 items that are rated on a Likert-type scale with a total composite score ranging from 17 to 85.^33^ A score of 44 or greater suggests a high likelihood for a PTSD clinical diagnosis, and a decrease of at least 10 points is considered a minimal clinically important improvement.^34^,^35^

The Generalized Anxiety Disorder 7-item scale (GAD-7) is a screening tool used in primary care for identifying anxiety within the last 2 weeks. Seven items are scored from 0 (not at all) to 3 (nearly every day) and total scores range from 0 to 21.^36^ Total scores suggest the severity of the anxiety: mild anxiety (5–9), moderate anxiety (10–14), and severe anxiety (15–21). Clinically, a score of 8 or higher is considered to warrant treatment, while a change of 4 to 5 points is considered clinically relevant.^37^

The Perceived Stress Scale (PSS), a 10-item survey measures the perception of stress within the last month. Items are designed to tap how unpredictable, uncontrollable, and overloaded respondents find their lives. Answers range from 0 (never) to 4 (very often), and the total scores range from 0 to 40. Categories for stress include low stress (0–13), moderate stress (14–26), and high perceived stress (27–40).^38^

To evaluate quality of life, the EQ-5D was used. This is a brief, standardized measure of current health status from the EuroQol Group that has 5 items with choices that range from 0 to 2. Total scores range from 0 to 10.^39^ There is also a single, self-reported index for health status (on a scale of 0–100).

#### Autonomic cardiovascular regulation

Due to the objective nature of this outcome measure, and previous studies demonstrating a positive effect of HIRREM on autonomic function, as well as reported disturbance of autonomic cardiovascular regulation among law enforcement personnel continuous recordings of blood pressure (BP) and heart rate (HR) were obtained. Recording was done following completion of symptom inventories and was performed with a noninvasive finger arterial pressure cuff and electrocardiogram for 10 minutes while the subject is resting supine and breathing freely. Files generated with systolic BP, beat-to-beat interval, and RR interval at 1000 Hz via the data acquisition system (BIOPAC acquisition system and Acknowledge 4.2 software, Santa Barbara, California) were analyzed with Nevrokard SA-BRS software (by Nevrokard Kiauta, d.o.o., Izola, Slovenia). The recordings were visually inspected to ensure data quality (dropped beats or gross motion artifacts are excluded), and the first 5 minutes of usable tracings were analyzed. These objective assessments included multiple measures of BP, HRV in both time and frequency domains, and BRS.

#### HIRREM intervention

An initial assessment (45 min) of brain electrical activity was conducted to obtain information about baseline patterns. The assessment consisted of 2-channel recordings from various locations on the scalp (F3/F4, C3/C4, T3/T4, P3/P4, FZ/OZ, O1/O2, FP1/FP2, and CB1/CB2) while the participant was at rest and carrying out a task. One-minute recordings were captured during the assessment at each location with eyes closed relaxing, eyes partially open, and eyes open performing a given mental task that corresponded with the area being observed (eg, recalling numbers, reading a passage, math problems). The information from the assessment informed the team regarding baseline balance between homologous brain regions and the distribution of electrical amplitudes.

The HIRREM intervention was delivered in a series of sessions that usually lasted between 90 and 120 minutes, as previously reported.^15^,^21^ Two sessions were completed in a half-day with a 20- to 30-minute break. Each session was comprised of 5 to 9 individualized protocols ranging from 8 to 30 minutes each. Each protocol was selected by a trained technologist based on the brain electrical activity data from the assessment or preceding session. The number of sessions for each participant was based on the brain pattern evolution across sessions relative to hemispheric symmetry and more optimal proportionation, subjective reports of clinical improvements, schedules, and preferences.

### Statistical Analysis

Two-tailed paired *t* tests were performed to evaluate mean comparison, pre- to post-HIRREM changes. There were no prior data available for this specific cohort by which to calculate sample size estimates. Due to the small sample size of this exploratory, pilot study, nonparametric Wilcoxon signed-rank test was performed to validate *t* test findings. Statistical tests were performed using SAS software version 9.4 (SAS Institute, Inc., Cary, North Carolina).

## Results

Twenty law enforcement personnel were screened, and 15 met eligibility criteria, provided informed consent, and completed the study. Of the 5 who were excluded, 4 never scheduled their sessions, and 1 was excluded for an ongoing need for medications. There were 14 men, and the group was largely Caucasian (13 Caucasian and 2 African American). The mean age of the cohort was 45.7 (SD = 5.6). Participants were sworn members and had been in law enforcement for an average of 23.2 (SD = 4.3) years. Eleven were part of the Winston-Salem Police Department, and 4 were part of the Forsyth County Sheriff’s Office. Years in service ranged from 14 to 28 years, and 13 were officers at the rank of Corporal or above. Five participants had previously served in the military. Table 1 highlights the self-reported health conditions.

**Table 1.** Self-Reported Health Conditions.

Condition	Number of Participants Reporting
Headaches	6
Hypertension	6
Insomnia	5
Migraines	5
Depression	4
Lipid disorder	4
Sports-related concussion	4
Stress/anxiety	4
Chronic fatigue	3
Chronic pain	3
Diabetes	2
Posttraumatic stress disorder	2
Tinnitus	2
Traumatic brain injury/head injury	2
Cancer	1
Hot flashes	1
Thyroid disorder	1
Vertigo/dizziness	1

The cohort received an average of 12.2 (SD = 2.7) HIRREM sessions over 7.9 (SD = 2.2) in-office days. Six of the 15 had at least 1 break in sessions due to scheduling conflicts (defined as having at least 5 days between any sessions). Data were collected at baseline, at V2 (22.8, SD = 9.2), and at V3 (67.2, SD = 14.1) days after intervention completion. There were no serious adverse events reported, no dropouts, and none were lost to follow up.

**Figure 1.**
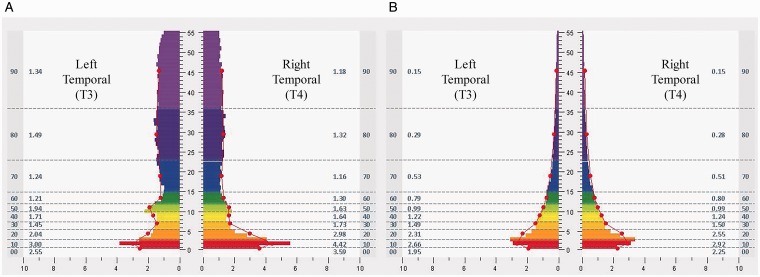
FFT Spectral Displays From a 40-Year-Old Male Participant, as an Example of Changes Observed in Electroencephalic Data, With Frequency (Hz, Central *Y*-Axis) Plotted Against Transformed Amplitude (µv, *X*-axis). Results represent 1 minute of data recorded from the T3/T4 montage with eyes closed at the baseline assessment (A) and the penultimate minute of the 15th session at the same location and eye state (B). Also note the reduced hyperarousal and improved balance.

Figure 1 demonstrates an example of brain pattern shifts seen during the intervention in 1 participant, a 40-year-old man with 22 years of service. At baseline, the pattern suggested hyperarousal in high frequencies at the temporal lobes, with eyes closed. The pattern during the penultimate minute of a protocol at the same location and eye state, in his penultimate session (15th), showed quieting, with improved proportionation and good balance.

**Figure 2.**
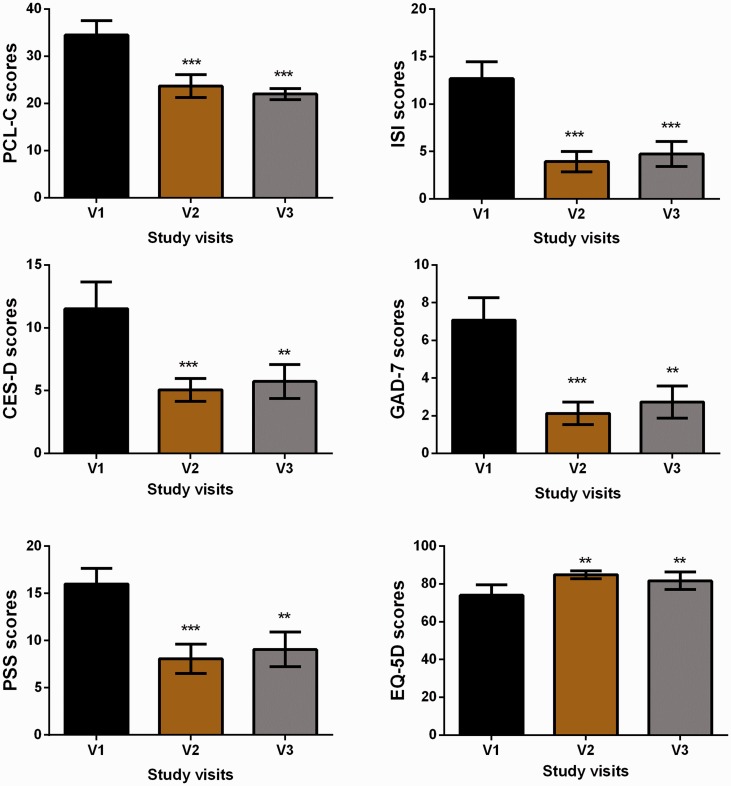
Scores for Symptom Inventories at the Baseline Study Visit (V1) and at Subsequent Follow-up Data Collection Visits (V2 and V3) Following the Use of a Closed-Loop Allostatic Neurotechnology. Symptom inventories include the PCL-C, ISI, CES-D, GAD-7, PSS, and EQ-5D. Error bars reflect the standard error of the mean. Statistical significance for changes between baseline and each follow-up visit is reflected as **P < .01; ***P ≤ .001. CES-D, Centers for Epidemiological Studies-Depression; EQ-5D, quality of life measure; GAD-7, Generalized Anxiety Disorder-7; ISI, Insomnia Severity Index; PCL-C, PTSD Checklist-Civilian version; PSS, Perceives Stress Scale.

Changes in symptom scores for insomnia, anxiety, stress, depressive mood, posttraumatic stress, and quality of life are shown in Figure 2. All self-reported symptom inventories had statistically significant reductions, with durability to V3, and those measures with established values for such showed clinically meaningful reductions. Table 2 details the changes by clinical category for each measure from pre to post-HIRREM. For insomnia, all subjects moved to having either none or subthreshold insomnia at V2 and V3. Categorical shifts to a higher percentage of participant scores being classified in the none/low category at V2 and V3 were seen for all symptom measures and were particularly noticeable for perceived stress and anxiety (both shifted from 4/15 at baseline to 11/15 at V3).

**Table 2.** Clinical Categories of Self-Report Scores.

Insomnia Severity Index	V1	V2	V3
0–7: No clinically significant insomnia	4	12	10
8–14: Subthreshold insomnia	5	3	5
15–21: Moderate severity clinical insomnia	5	0	0
22–28: Severe clinical insomnia	1	0	0
Generalized Anxiety Disorder-7	V1	V2	V3
0–4: No anxiety	4	12	11
5–7: Mild anxiety	7	3	2
10–14: Moderate anxiety	3	0	2
15–21: Severe anxiety	1	0	0
Perceived Stress Scale	V1	V2	V3
0–13: Low stress	4	13	11
14–26: Moderate stress	11	2	4
27–40: High perceived stress	0	0	0
Center for Epidemiologic Studies Depression scale	V1	V2	V3
0–15: No depression	11	15	14
16–60: Clinical depression	4	0	1
Posttraumatic Stress Disorder Checklist	V1	V2	V3
17–43: No PTSD	13	14	15
44–85: Symptoms suggesting PTSD	2	1	0

Abbreviation: PTSD, posttraumatic stress disorder.

**Figure 3.**
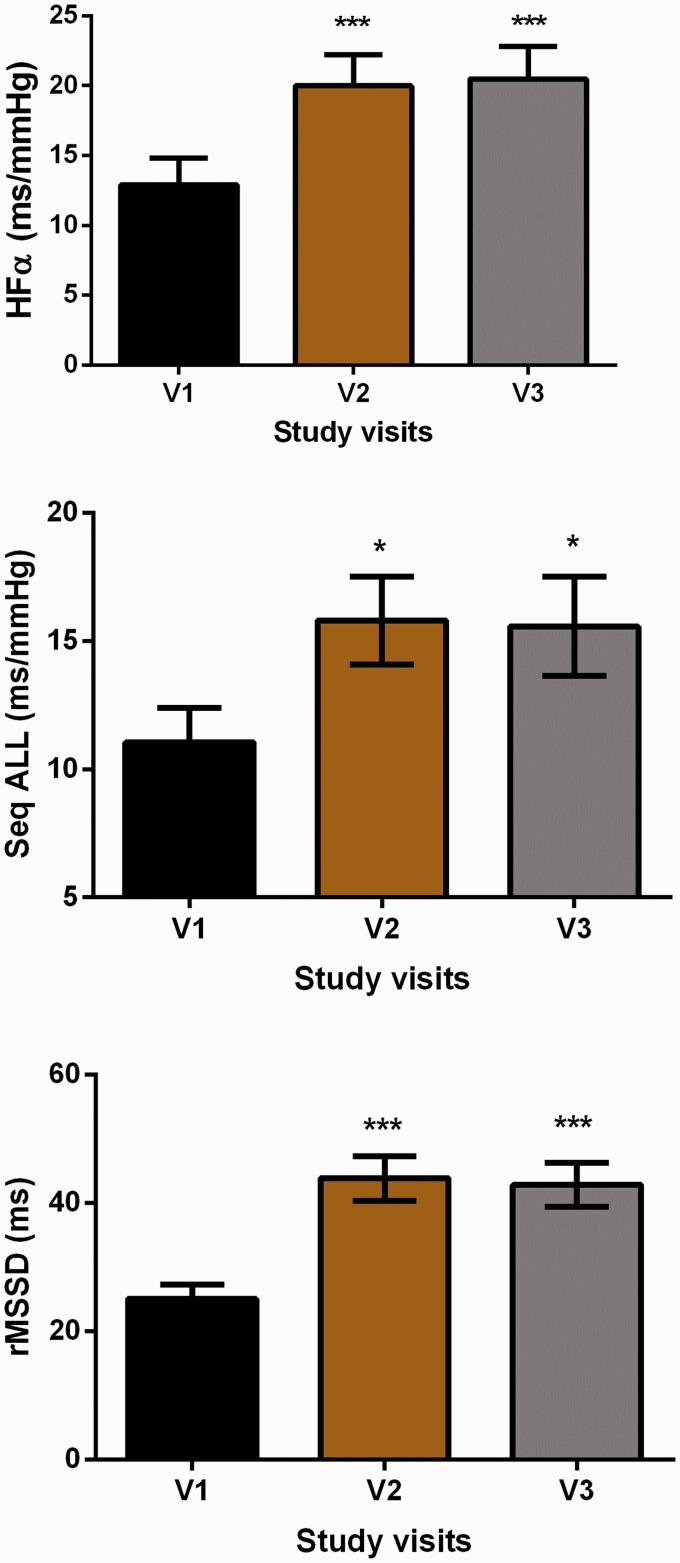
Values for Heart Rate Variability, Baroreflex Sensitivity, and Blood Pressure, Before and After Intervention. Error bars are standard error of the mean. *P < .05; **P < .01; ***P < .001 Versus Visit 1 (V1). HFα, high-frequency alpha; rMSSD, root mean square of the successive differences; Seq ALL, sequence all for change in RR interval for changes in blood pressure; V1, baseline study visit; V2, immediately after intervention completion.

Analysis of autonomic cardiovascular regulation showed significant improvements (increases) in multiple measures of HRV and BRS, as evidenced by increased values for the root mean square of the successive differences (rMSSD; delta V1–V2 = 18.7 ms [SD = 17.1], *P* =.0004; delta V1–V3 = 17.7 ms [15.2], *P* = .001), high-frequency alpha (HF alpha; delta V1–V2 = 12.9 ms/mm Hg [7.1], *P* = .0006; delta V1–V3 = 7.5 ms/mm Hg [9.9], *P* = .0003), and sequence ALL (delta V1–V2 =4.7 ms/mm Hg [6.0], *P* = .01; delta V1–V3 = 4.5 ms/mm Hg [5.9], *P* = .01; Figure 3).

## Discussion

Chronic stress and sleep disruption experienced by those in law enforcement is associated with increased risk for a variety of adverse health outcomes.^1^,^2^,^8^ Although most assume that acute incidents are the main culprit, stress in law enforcement can be due to a variety of organizational, operational, external, and personal factors.^40^ Irrespective of the causes, the need for therapeutic strategies to reduce symptoms, improve sleep, and improve autonomic balance and function is clear.

This study evaluated changes in a variety of self-reported behavioral symptoms, and autonomic cardiovascular regulation, associated with the use of HIRREM in a sworn law enforcement cohort. There were significant improvements from baseline to post-HIRREM follow-up visits for self-reported symptoms of insomnia, depression, traumatic stress, perceived stress, anxiety, and quality of life. There was also improvement in objective measures of autonomic function as measured by HRV and BRS. The autonomic changes appear to primarily reflect increased parasympathetic tone, resulting in greater flexibility and dynamic range for autonomic responses. The benefits for both symptom reductions and improved autonomic function were durable to just over 2 months following completion of HIRREM.

The impact of stress with law enforcement is well recognized, and many therapeutic strategies have been evaluated. A Cochrane review reported on 10 studies that tested psychosocial interventions for police, including physical exercise, as potential ways to prevent psychological disorders including stress-related symptomatology. The authors concluded that “There is lack of evidence that psychosocial interventions can reduce stress-related psychological symptoms.”^41^ They noted that only one of the studies was focused on primary prevention (intervention to prevent the onset of disease), and that the studies were focused on the stress of acute incidents, as opposed to organizational, continuous, and perhaps more consequential stresses of the general law enforcement work environment.

Others have since reported on the use of a variety of team-based, individual, cognitive, and physiological-based approaches. Kuehl et al. studied the effectiveness of a team-based strategy. The intervention consisted of a series of scripted, peer-led sessions on healthy eating, exercise, body weight, stress, sleep, and other lifestyle behaviors. Benefits were seen at 6 months;^42^ but at the 12-month follow-up, only the differences for healthy eating habits remained.^43^

Using a cognitive strategy with a focus on mental imagery, Arnetz et al. found that police cadets (n = 37) who participated in a 9-week resilience training course reported better general health and more problem-based coping skills. This persisted through 2 years of follow-up, compared to a control group (n = 38) who did not take the course.^44^ Eight weeks of a Mindfulness-Based Resilience Training program resulted in initial reductions in salivary cortisol, self-reported aggression, organization stress, burnout, sleep disturbance, but was not maintained at the 3-month follow-up.^45^ A brief, group-based fatigue management training program also improved self-reported insomnia at 4 weeks, but did not affect other behavioral symptoms, and there was no evaluation of impact on autonomic function.^46^

McCraty and Atkinson reported on their controlled study to promote resilience in police officers through emotional management training and HRV biofeedback (HeartMath^®^), provided over 3 separate classroom sessions lasting 4 hours each.^47^ Postinterventional differences between the groups were mostly nonstatistically significant, and there were no stated changes in physiological measures that could be attributed to the training. Other studies using variants of the HeartMath technique have shown modest changes in nonrandomized designs.^48–50^

Limitations of our study, which preclude making definitive conclusions, include the small sample size, and a single-arm, open label design. In addition, the beneficial effect of participating in a clinical research study as well as the impact of therapeutic expectation cannot be adequately evaluated in this study. In a previous controlled clinical trial of HIRREM for insomnia, expectation effect did not result in improved autonomic function for the control group who received random tones not linked to brainwaves. However, significant improvement was observed in the active intervention group, as was seen in the current cohort. This suggests that measurable, persisting physiological shifts occurred. This cohort consisted of all sworn personnel, virtually all of whom were officers, reflecting potential recruitment bias, so generalizability to patrolmen and nonsworn personnel cannot be assessed. A longer period of follow-up to better evaluate durability would be desirable.

## Conclusion

Stress and sleep disruption adversely affect health and performance in law enforcement as well as first responders, and there is a pressing need for more effective therapies. These pilot data provide the first report of both significantly reduced symptom and improved objective measures of autonomic cardiovascular regulation, associated with the use of HIRREM in a series of sworn law enforcement personnel. Sessions were well tolerated, with no dropouts, or serious adverse events. These results are consistent with those previously reported regarding the use of HIRREM for other symptoms and conditions. They also provide proof of concept for a brain-focused intervention to address issues of stress and insomnia with law enforcement. Further studies are warranted, using designs that can better address potential limitations.

## References

[1] Violanti JM Hartley TA Gu JK Fekedulegn D Andrew ME Burchfiel CM. Life expectancy in police officers: a comparison with the U.S. general population. Int J Emerg Ment Health. 2013; 15(4):217–228. 24707585 PMC4734369

[2] Zimmerman FH. Cardiovascular disease and risk factors in law enforcement personnel: a comprehensive review. Cardiol Rev. 2012; 20(4):159–166. 22314143 10.1097/CRD.0b013e318248d631

[3] Juster RP Sindi S Marin MF, et al A clinical allostatic load index is associated with burnout symptoms and hypocortisolemic profiles in healthy workers. Psychoneuroendocrinology. 2011; 36(6):797–805. 21129851 10.1016/j.psyneuen.2010.11.001

[4] Sinha SS. Trauma-induced insomnia: a novel model for trauma and sleep research. Sleep Med Rev. 2016; 25:74–83. 26140870 10.1016/j.smrv.2015.01.008

[5] Gehrman P Seelig AD Jacobson IG, et al Predeployment sleep duration and insomnia symptoms as risk factors for new-onset mental health disorders following military deployment. Sleep. 2013; 36(7):1009–1018. 23814337 10.5665/sleep.2798 PMC3669076

[6] Hartley TA Burchfiel CM Fekedulegn D Andrew ME Violanti JM. Health disparities in police officers: comparisons to the U.S. general population. Int J Emerg Ment Health. 2011; 13(4):211–220. 22900455 PMC4734372

[7] Rajaratnam SW Barger LK Lockley SW, et al Sleep disorders, health, and safety in police officers. JAMA. 2011; 306(23):2567–2578. 22187276 10.1001/jama.2011.1851

[8] Mumford EA Taylor BG Kubu B. Law enforcement officer safety and wellness. Police Q. 2015; 18(2):111–133.

[9] McEwen BS. Protective and damaging effects of stress mediators. N Engl J Med. 1998; 338(3):171–179. 9428819 10.1056/NEJM199801153380307

[10] Porges SW. The polyvagal perspective. Biol Psychol. 2007; 74(2):116–143. 17049418 10.1016/j.biopsycho.2006.06.009 PMC1868418

[11] La Rovere MT Bigger JT Jr. Marcus FI Mortara A Schwartz PJ. Baroreflex sensitivity and heart-rate variability in prediction of total cardiac mortality after myocardial infarction. ATRAMI (Autonomic Tone and Reflexes After Myocardial Infarction) Investigators. The Lancet. 1998; 351(9101):478–484. 10.1016/s0140-6736(97)11144-8 9482439

[12] Thayer JF Hansen AL Saus-Rose E Johnsen BH. Heart rate variability, prefrontal neural function, and cognitive performance: the neurovisceral integration perspective on self-regulation, adaptation, and health. Ann Behav Med. 2009; 37(2):141–153. 19424767 10.1007/s12160-009-9101-z

[13] Charles LE Andrew ME Sarkisian K, et al Associations between insulin and heart rate variability in police officers. Am J Hum Biol. 2014; 26(1):56–63. 24136902 10.1002/ajhb.22475 PMC4657740

[14] Andrew ME Violanti JM Gu JK, et al Police work stressors and cardiac vagal control. Am J Hum Biol. 2017; 29(5):e22996. 10.1002/ajhb.22996 PMC6314196 28295842

[15] Gerdes L Gerdes P Lee SW Tegeler CH. HIRREM: a noninvasive, allostatic methodology for relaxation and auto-calibration of neural oscillations. Brain Behav. 2013; 3(2):193–205. 23532171 10.1002/brb3.116 PMC3607159

[16] Tegeler CH Kumar SR Conklin D, et al Open label, randomized, crossover pilot trial of high-resolution, relational, resonance-based, electroencephalic mirroring to relieve insomnia. Brain Behav. 2012; 2(6):814–824. 23170244 10.1002/brb3.101 PMC3500468

[17] Tegeler CH Tegeler CL Cook JF Lee SW Pajewski NM. Reduction in menopause-related symptoms associated with use of a noninvasive neurotechnology for autocalibration of neural oscillations. Menopause. 2015; 22(6):650–655. 25668305 10.1097/GME.0000000000000422 PMC4448674

[18] Tegeler CH Tegeler CL Cook JF, et al A preliminary study of the effectiveness of an allostatic, closed-loop, acoustic stimulation neurotechnology in the treatment of athletes with persisting post-concussion symptoms. Sports Med Open. 2016; 2(1):39. 27747793 10.1186/s40798-016-0063-y PMC5023638

[19] Tegeler CH Cook JF Tegeler CL, et al Clinical, hemispheric, and autonomic changes associated with use of closed-loop, allostatic neurotechnology by a case series of individuals with self-reported symptoms of post-traumatic stress. BMC Psychiatry. 2017; 17(1):141. 28420362 10.1186/s12888-017-1299-x PMC5395741

[20] Tegeler CL Gerdes L Shaltout HA, et al Successful use of closed-loop allostatic neurotechnology for post-traumatic stress symptoms in military personnel: self-reported and autonomic improvements. Mil Med Res. 2017; 4(1):38. 29502530 10.1186/s40779-017-0147-0 PMC5740870

[21] Shaltout HA Lee SW Tegeler CL, et al Improvements in heart rate variability, baroreflex sensitivity, and sleep after use of closed-loop allostatic neurotechnology by a heterogeneous cohort. Front Public Health. 2018; 6:116. 29922641 10.3389/fpubh.2018.00116 PMC5996903

[22] Tegeler CH Shaltout HA Tegeler CL Gerdes L Lee SW. Rightward dominance in temporal high-frequency electrical asymmetry corresponds to higher resting heart rate and lower baroreflex sensitivity in a heterogeneous population. Brain Behav. 2015; 5(6):e00343. 26085968 10.1002/brb3.343 PMC4467777

[23] Lee SW Laurienti PJ Burdette JH, et al Functional brain network changes following use of an allostatic, closed-loop, acoustic stimulation neurotechnology for military-related traumatic stress. J Neuroimaging. 2019; 29(1):70–78. 30302866 10.1111/jon.12571 PMC6586033

[24] Tegeler CL Shaltout HA Howard LJ Schmidt KD Garlin EI Tegeler CH. Abstract P484: improved heart rate variability, and symptoms of insomnia and stress, with use of a closed-loop, allostatic neurotechnology in law enforcement officers. Hypertension. 2017; 70(suppl 1):AP484–AP484.

[25] Shaltout HA Tegeler CL Tegeler CH. Abstract P485: use of a noninvasive, closed-loop, allostatic, neurotechnology reduced blood pressure and improved heart rate variability in a pre-hypertensive cohort. Hypertension. 2017; 70(suppl 1):AP485–AP485.

[26] Morin CM Barlow DH Dement WC. Insomnia: Psychological Assessment and Management. New York, NY: Guilford Press; 1993.

[27] Morin CM Belleville G Belanger L Ivers H. The Insomnia Severity Index: psychometric indicators to detect insomnia cases and evaluate treatment response. Sleep. 2011; 34(5):601–608. 21532953 10.1093/sleep/34.5.601 PMC3079939

[28] Yang M Morin CM Schaefer K Wallenstein GV. Interpreting score differences in the Insomnia Severity Index: using health-related outcomes to define the minimally important difference. Curr Med Res Opin. 2009; 25(10):2487–2494. 19689221 10.1185/03007990903167415

[29] Bastien CH Vallieres A Morin CM. Validation of the Insomnia Severity Index as an outcome measure for insomnia research. Sleep Med. 2001; 2(4):297–307. 11438246 10.1016/s1389-9457(00)00065-4

[30] Radloff LS. The CES- D scale: A self-report depression scale for research in the general population. Appl Psychol Meas. 1977; 1(3):385–401.

[31] Smarr KL. Measures of depression and depressive symptoms: the Beck depression inventory (BDI), Center for Epidemiological Studies-Depression Scale (CES-D), geriatric depression scale (GDS), hospital anxiety and depression scale (HADS), and primary care evaluation of mental disorders-mood module (PRIME-MD). Arthritis Care Res. 2003; 49:S134–S146. 10.1002/acr.20556 22588766

[32] American Psychiatric Association. Diagnostic and Statistical Manual of Mental Disorders, 4th ed., Text Revision. Washington, DC: American Psychiatric Association; 2000.

[33] Weathers FW Litz BT Herman DS Huska JA Keane TM. The PTSD Checklist (PCL): reliability, validity, and diagnostic utility. Paper presented at: 9th Annual Meeting of the International Society for Traumatic Stress Studies; 1993; San Antonio, TX.

[34] Blanchard EB Jones-Alexander J Buckley TC Forneris CA. Psychometric properties of the PTSD Checklist (PCL). Behav Res Ther. 1996; 34(8):669–673. 8870294 10.1016/0005-7967(96)00033-2

[35] Monson CM Gradus JL Young-Xu Y Schnurr PP Price JL Schumm JA. Change in posttraumatic stress disorder symptoms: do clinicians and patients agree? Psychol Assess. 2008; 20(2):131–138. 18557690 10.1037/1040-3590.20.2.131

[36] Spitzer RL Kroenke K Williams JB Lowe B. A brief measure for assessing generalized anxiety disorder: the GAD-7. Arch Intern Med. 2006; 166(10):1092–1097. 16717171 10.1001/archinte.166.10.1092

[37] Plummer F Manea L Trepel D McMillan D. Screening for anxiety disorders with the GAD-7 and GAD-2: a systematic review and diagnostic metaanalysis. Gen Hosp Psychiatry. 2016; 39:24–31. 26719105 10.1016/j.genhosppsych.2015.11.005

[38] Cohen S Kamarck T Mermelstein R. A global measure of perceived stress. J Health Soc Behav. 1983; 24(4):385–396. 6668417

[39] Rabin R de CF. EQ-5D: a measure of health status from the EuroQol Group. Ann Med. 2001; 33(5):337–343. 11491192 10.3109/07853890109002087

[40] Dempsey JS Forst LS. An Introduction to Policing. Boston, MA: Cengage Learning; 2013.

[41] Penalba V McGuire H Leite JR. Psychosocial interventions for prevention of psychological disorders in law enforcement officers. Cochrane Database Syst Rev. 2008; 3:CD005601. 10.1002/14651858.CD005601.pub2 18646132

[42] Kuehl KS Elliot DL Goldberg L, et al The safety and health improvement: enhancing law enforcement departments study: feasibility and findings. Front Public Health. 2014; 2:38. 24847475 10.3389/fpubh.2014.00038 PMC4021110

[43] Kuehl KS Elliot DL MacKinnon DP, et al The SHIELD (Safety & Health Improvement: Enhancing Law Enforcement Departments) study: mixed methods longitudinal findings. J Occup Environ Med. 2016; 58(5):492–498. 27158956 10.1097/JOM.0000000000000716 PMC4863458

[44] Arnetz BB Arble E Backman L Lynch A Lublin A. Assessment of a prevention program for work-related stress among urban police officers. Int Arch Occup Environ Health. 2013; 86(1):79–88. 22366986 10.1007/s00420-012-0748-6 PMC3596819

[45] Christopher Michael S Hunsinger M Goerling LRichard J, et al Mindfulness-based resilience training to reduce health risk, stress reactivity, and aggression among law enforcement officers: a feasibility and preliminary efficacy trial. Psychiatry Res. 2018; 264:104–115. 29627695 10.1016/j.psychres.2018.03.059 PMC6226556

[46] James L Samuels CH Vincent F. Evaluating the effectiveness of fatigue management training to improve police sleep health and wellness: a pilot study. J Occup Environ Med. 2018; 60(1):77–82. 28953073 10.1097/JOM.0000000000001174

[47] McCraty R Atkinson M. Resilience training program reduces physiological and psychological stress in police officers. Glob Adv Health Med. 2012; 1(5):44–66. 27257532 10.7453/gahmj.2012.1.5.013 PMC4890098

[48] Weltman G Lamon J Freedy E Chartrand D. Police department personnel stress resilience training: an institutional case study. Glob Adv Health Med. 2014; 3(2):72–79. 24808985 10.7453/gahmj.2014.015 PMC4010956

[49] Ramey SL Perkhounkova Y Hein M Chung S Franke WD Anderson AA. Building resilience in an urban police department. J Occup Environ Med. 2016; 58(8):796–804. 27414008 10.1097/JOM.0000000000000791

[50] Andersen JP Papazoglou K Koskelainen M Nyman M Gustafsberg H Arnetz BB. Applying resilience promotion training among special forces police officers. SAGE Open. 2015; 5(2):1–14. 10.1177/2158244015590446 PMC4484868 26137394

